# Supplementary material for: Evidence supported by Mendelian randomization: impact on inflammatory factors in knee osteoarthritis
Source: Front Med (Lausanne). 2024 May 28;11:1382836. doi: 10.3389/fmed.2024.1382836 (PMC11165061; doi:10.3389/fmed.2024.1382836)
Supplement: Supplementary file 1 [file Data_Sheet_1.ZIP › data/GCST90274803_5e6/803.leaveoneout.pdf]

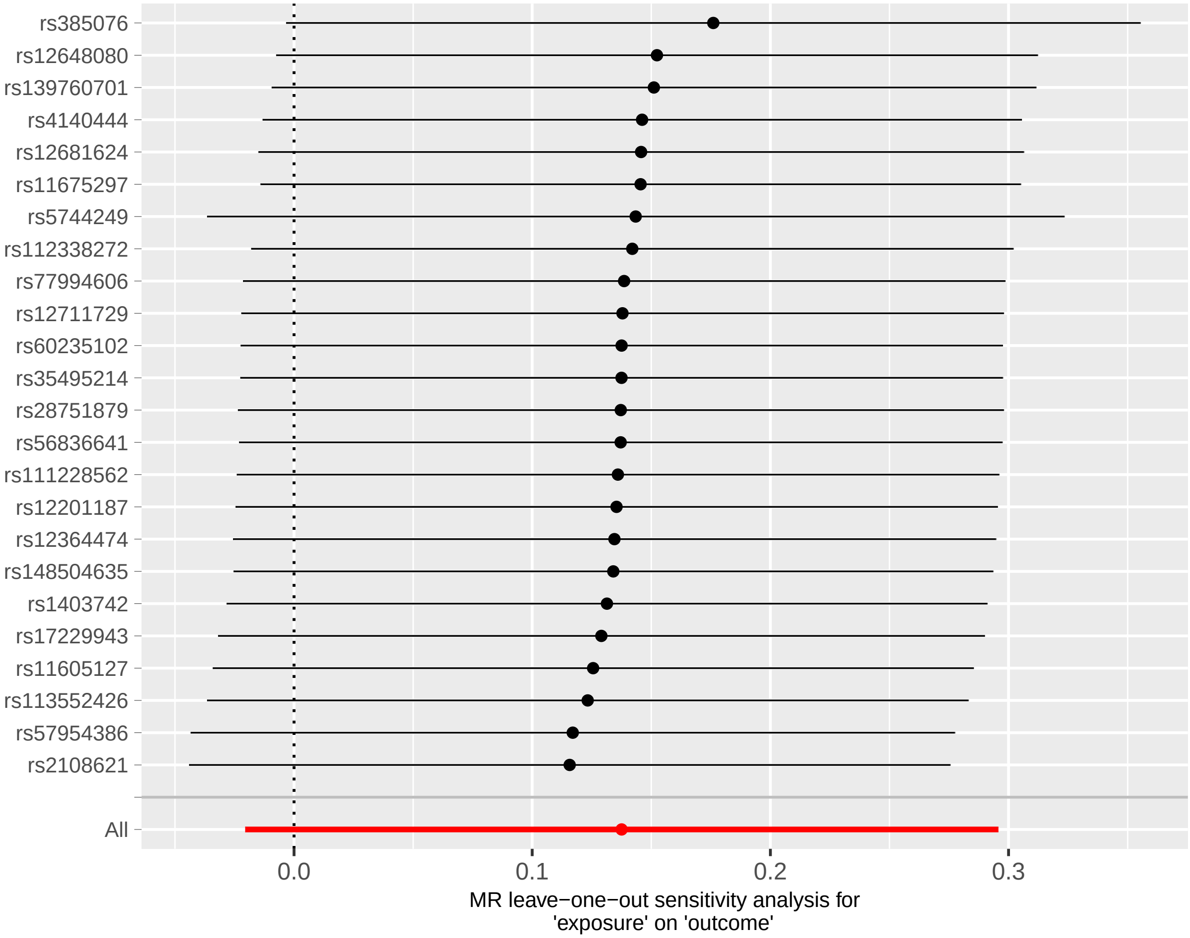
rs385076
rs12648080
rs139760701
rs4140444
rs12681624
rs11675297
rs5744249
rs112338272
rs77994606
rs12711729
rs60235102
rs35495214
rs28751879
rs56836641
rs111228562
rs12201187
rs12364474
rs148504635
rs1403742
rs17229943
rs11605127
rs113552426
rs57954386
rs2108621
All
0.0
0.1
0.2
0.3
MR leave−one−out sensitivity analysis for
'exposure' on 'outcome'
